# Epigenetics-related genes in prostate cancer: Expression profile in prostate cancer tissues, androgen-sensitive and -insensitive cell lines

**DOI:** 10.3892/ijmm.2012.1173

**Published:** 2012-11-06

**Authors:** DAVID ADLER, ANDREAS LINDSTROT, JACQUELINE OCHSENFAHRT, KERSTIN FUCHS, NICOLAS WERNERT

**Affiliations:** Institute of Pathology, University Hospital Bonn, D-53127 Bonn, Germany

**Keywords:** epigenetics, prostate cancer, cell lines

## Abstract

Epigenetic changes have been suggested to drive prostate cancer (PCa) development and progression. Therefore, in this study, we aimed to identify novel epigenetics-related genes in PCa tissues, and to examine their expression in metastatic PCa cell lines. We analyzed the expression of epigenetics-related genes via a clustering analysis based on gene function in moderately and poorly differentiated PCa glands compared to normal glands of the peripheral zone (prostate proper) from PCa patients using Whole Human Genome Oligo Microarrays. Our analysis identified 12 epigenetics-related genes with a more than 2-fold increase or decrease in expression and a p-value <0.01. In moderately differentiated tumors compared to normal glands of the peripheral zone, we found the genes, *TDRD1*, *IGF2*, *DICER1*, *ADARB1*, *HILS1*, *GLMN* and *TRIM27,* to be upregulated, whereas *TNRC6A* and *DGCR8* were found to be downregulated. In poorly differentiated tumors, we found *TDRD1*, *ADARB* and *RBM3* to be upregulated, whereas *DGCR8*, *PIWIL2* and *BC069781* were downregulated. Our analysis of the expression level for each gene in the metastatic androgen-sensitive VCaP and LNCaP, and -insensitive PC3 and DU-145 PCa cell lines revealed differences in expression among the cell lines which may reflect the different biological properties of each cell line, and the potential role of each gene at different metastatic sites. The novel epigenetics-related genes that we identified in primary PCa tissues may provide further insight into the role that epigenetic changes play in PCa. Moreover, some of the genes that we identified may play important roles in primary PCa and metastasis, in primary PCa only, or in metastasis only. Follow-up studies are required to investigate the functional role and the role that the expression of these genes play in the outcome and progression of PCa using tissue microarrays.

## Introduction

Prostate cancer (PCa) is one of the most common types of cancer affecting males in Western countries (1). Both steadily increasing age and the introduction of the serum marker prostate-specific antigen (PSA) have led to a steady increase in PCa incidence among Western populations (2,3). The course of PCa is highly variable, from indolent carcinomas with a favorable prognosis requiring only watchful waiting, to highly lethal aggressive carcinomas with distant metastases needing systemic treatment (4,5). Unfortunately, it is not possible to predict precisely based upon clinical data and biopsy alone the outcome of PCa in individual patients (3), as the disease is clinically heterogeneous and often multifocal with a clinical outcome that is difficult to predict (6,7). As a result, tremendous efforts and resources have been invested over the years with the ultimate aim of identifying molecular markers possessing independent prognostic clues or serving as potential therapeutic targets in PCa.

Similar to other cancers, the development and progression of PCa is driven by the interplay of genetic as well as epigenetic changes (5). Epigenetic changes result in the modification of gene expression and function without changes in the DNA sequence (8). They include both the direct methylation of the CpG islands (9–11) and histone modifications (e.g., acetylation, ADP-ribosylation, ubiquination, phosphorylation and methylation) (9,11). The extent of histone methylation may vary (mono- to trimethylation) and the location of the modification can determine whether a gene will be silenced or transcribed (9). Both DNA methylation and histone modifications have been implicated in PCa, and have been proposed to influence tumor formation (11). For example, more than 50 hypermethylated genes have been found in PCa, and approximately a dozen of these genes have been found to be consistently affected in the majority of cases examined (5). Many of the genes that have been found to be methylated in PCa are known to affect cell cycle control, tumor invasion and signal transduction (11).

Due to the crucial role that epigenetic changes play in PCa and their potential use as diagnostic and prognostic markers in the future, we in this study, aimed to identify novel epigenetics-related genes in primary PCa tissues, and to examine their expression in metastatic androgen-sensitive and -insensitive PCa cell lines.

## Materials and methods

### Ethics statement

This study was approved by the Ethics Eeview Board of the University of Bonn/University Hospital, Bonn, Germany, according to the principles expressed in the Declaration of Helsinki. Written informed consents were obtained from all participants.

### Microarray raw data

The microarray raw data were deposited in the GEO database under accession number: GSE28615 and we confirm that all details are MIAME compliant.

### Processing of human prostatectomy specimens

Prostate tissue samples were obtained from PCa patients immediately after radical prostatectomy. Fresh tissue samples (0.5×0.5×0.3 cm) were obtained from the peripheral zones (prostate proper) of 5 moderately differentiated (Gleason scores 6 and 7a) PCa patients, 4 poorly differentiated (Gleason scores 8 and 9) PCa patients, as well as from 10 normal peripheral zones of PCa patients. The tissues were shock-frozen in liquid nitrogen with ice-cold isopentane. Frozen sections (6-μm thick) were cut from the samples using a cryotome (Leica, Germany) and mounted on membrane-coated slides (Membrane Slides, 1 mm PEN, Zeiss, Jena, Germany) for subsequent laser microdissection. One section was mounted on conventional slides and stained with hematoxylin and eosin (H&E) for diagnostic evaluation by an experienced pathologist. Laser microdissection was performed as previously described (12–14). The frozen sections were then dried for 2 min in the cryotome, washed for 2 min with 70% ethanol in DEPC-treated water and stained for 30 sec in 1% cresyl violet diluted in 50% ethanol-DEPC-treated water. The slides were then washed briefly in 70 and 100% ethanol, dried for 10 min and stored at −80˚C until use for laser microdissection of normal glands and the stroma between them.

### Quality control

RNA quality was measured from each patient before laser microdissection using laser capture microscopy (LCM). The section was washed from the slide with 600 μl buffer RLT + 2 μM DTT (RNeasy Mini kit, Qiagen, Hilden, Germany) and vortexed for 30 sec. RNA extraction was performed as described by the manufacturer. The recommended DNase digestion was made with RNase-free DNase set (Qiagen). RNA quality was measured with the Agilent Bioanalyzer 2100 (Agilent Technologies, Santa Clara, CA, USA). Samples with a RIN factor >6 were used for LCM.

### LCM

The cresyl violet-stained sections were visualized with an Axio Observer.Z1 microscope (Zeiss) with an installed Palm MicroBeam (Zeiss). LCM was performed under ×10 objective lenses. The glands from carcinomas and the normal peripheral zones were isolated from the stroma by laser microdissection and collected in 200-μl adhesive cap tubes (Zeiss).

### Microarray analysis of RNA isolated from laser-microdisected moderately and poorly differentiated prostate carcinoma glands, as well as from normal glands of PCa patients

RNA was isolated from laser-microdisected moderately and poorly differentiated PCa glands, and from normal glands from PCa patients using the RNeasy Micro kit (Qiagen) as described by the manufacturer. The recommend DNase digestion was included with the RNase-free DNase set (Qiagen). The amount of isolated RNA was measured with the Nanodrop photometer (Thermo Fisher Scientific, Hudson, NH, USA). Thereafter, an equal amount of RNA from the normal peripheral glands, moderately differentiated glands and poorly differentiated glands was pooled to final concentrations of 300 ng of RNA, respectively. These pools were then sent to Miltenyi Biotec (Bergisch Gladbach, Germany) for the microarray analysis and bioinformatics interpretation. The RNA was labelled with Cy3 and hybridized on the Whole Human Genome Oligo Microarray 4×44K (Agilent Technologies) according to the manufacturer’s instructions. The microarray results were then validated by qRT-PCR of a subset of genes.

### RNA isolation from PCa cell lines and cDNA synthesis

RNA was isolated from the PCa cell lines, VCaP, DU-145, LNCaP and PC3 using the RNeasy mini kit (Qiagen) as described by the manufacturer. The amount of isolated RNA was then measured with the ND1000 Nanodrop (Peqlab, Wilmington, DE, USA). Reverse transcription of total RNA was performed with the SuperScript III First-Strand Synthesis SuperMix as described by the manufacturer (Invitrogen, Carlsbad, CA, USA).

### Polymerase chain reaction (PCR)

PCR was performed using the DNA Engine Peltier thermal cycler (Bio-Rad, Munich, Germany) to analyze the expression of *RBM3*, *GLMN*, *BC068781*, *TNRC6A*, *TDRD1*, *DGCR8*, *HILS1*, *TRIM27*, *DICER1*, *PIWIL2*, *IGF2* and *ADARB1* and the housekeeping gene, *GAPDH*. The primers and sequences for all the latter genes are listed in Table I. All PCR products were analyzed on 2% agarose gels that were stained with ethidium bromide.

## Results

### Gene expression analysis of moderately and poorly differentiated prostate carcinoma glands compared to normal glands of the the peripheral zone (prostate proper) from PCa patients identifies epigenetics-related genes using Whole Human Genome Oligo Microarrays

To identify epigenetics-related genes in PCa, we analyzed the expression profile of epigenetics-related genes in the moderately and poorly differentiated PCa glands compared to normal glands of the peripheral zone (prostate proper) from PCa patients using 2 Whole Human Genome Oligo Microarrays. Genes with a more than 2-fold increase or decrease in expression and a p-value <0.01 were considered significant. A clustering analysis based on gene function identified 12 epigenetics-related genes (Tables II–V).

The epigenetics-related genes, *TDRD1*, *IGF2*, *DICER1*, *ADARB1*, *HILS1*, *GLMN* and *TRIM27,* were found to be upregulated in the moderately differentiated tumors compared to the normal glands of the peripheral zone (Table II), whereas TNRC6A and DGCR8 were found to be downregulated (Table III).

In the poorly differentiated tumors compared to the normal glands of the peripheral zone, the epigenetics-related genes, *TDRD1 ADARB* and *RBM3,* were found to be upregulated (Table IV), whereas *DGCR8*, *PIWIL2* and *BC069781* were found to be downregulated (Table V).

### Gene expression of epigenetics-related genes identified from PCa tissues in androgen-sensitive and -insensitive PCa cell lines

**Figure 1:**
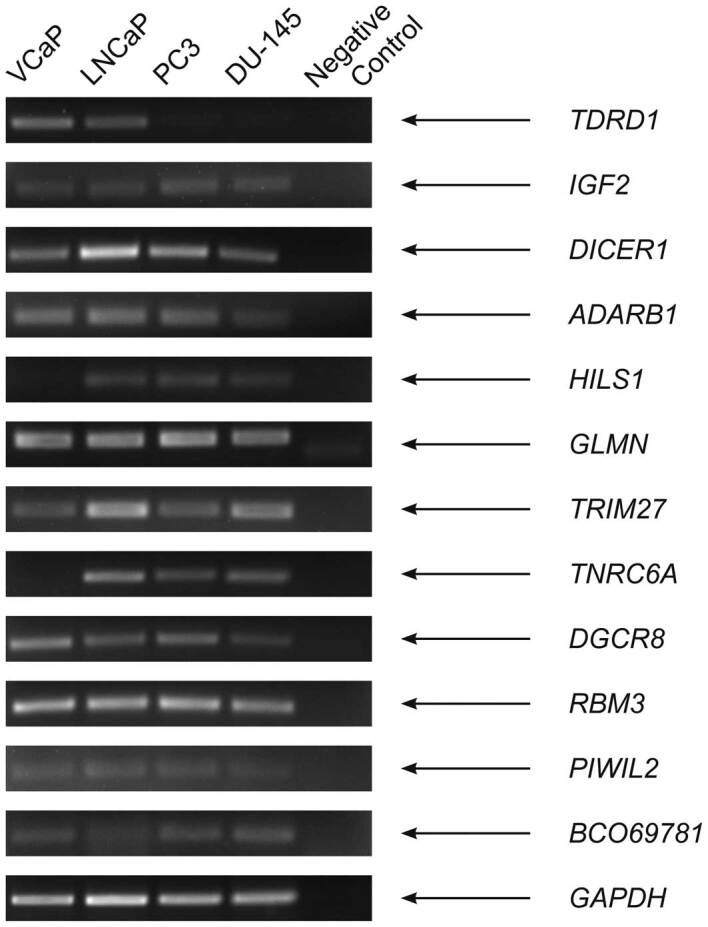
mRNA expression of epigenetics-related genes in prostate cancer cell lines. RT-PCR analysis of epigenetics-related genes in the androgen-dependent VCaP and LNCaP, and the androgen-independent PC3 and DU-145 prostate cancer cell lines. The gene *GAPDH* was used as the loading control, and amplification without a template was used as the negative control.

The epigenetics-related genes identified in PCa tissues (Tables II–V) were examined for their expression in the androgen-sensitive VCaP and LNCaP and -insensitive PC3 and DU-145 PCa cell lines (Fig. 1). The expression level for each gene differed among the 4 cell lines examined. For instance, *TDRD1* was expressed only in the androgen-sensitive PCa cell lines, but not in the insensitive ones. *HILS1* and *TNRC6A* were expressed in all the cell lines expect in the androgen-sensitive VCaP cell line. We found *PIWIL2* and *BCO69781* to be very weakly expressed in all the cell lines examined, whereas *IGF2*, *DICER1*, *ADARB1*, *GLMN*, *TRIM27*, *DGCR8* and *RBM3* were expressed in all 4 cell lines at different levels.

## Discussion

Genetic and epigenetic changes have been suggested to drive PCa development and progression (5). For instance, DNA methylation and histone modifications have been suggested to influence PCa tumor formation (11). Hypermethylated genes have been found in PCa (5), and many of them are known to affect various cellular processes, such as cell cycle control, tumor invasion and signal transduction (11). Therefore, due to the crucial role that epigenetic changes play in PCa and their potential use as diagnostic and prognostic markers in the future, in this study, we aimed to identify novel epigenetics-related genes in PCa tissues, and to examine their expression in metastatic androgen-sensitive and -insensitive PCa cell lines.

To identify epigenetics-related genes in PCa, we analyzed the expression profile of epigenetics-related genes via a clustering analysis based on gene function in the moderately and poorly differentiated PCa glands compared to normal glands of the peripheral zone (prostate proper) from PCa patients using Whole Human Genome Oligo Microarrays. Our analysis identified 12 epigenetics-related genes with a more than 2-fold increase or decrease in expression and a p-value <0.01 (Tables II–V).

In the moderately differentiated tumors compared to normal glands of the peripheral zone, we found the epigenetics-related genes, *TDRD1*, *IGF2*, *DICER1*, *ADARB1*, *HILS1*, *GLMN* and *TRIM27,* to be upregulated (Table II), whereas *TNRC6A* and *DGCR8* were found to be downregulated (Table III). In poorly differentiated tumors, we found *TDRD1*, *ADARB* and *RBM3* to be upregulated (Table IV), whereas *DGCR8*, *PIWIL2* and *BC069781* were downregulated (Table V).

Our literature search of these epigenetics-related genes revealed 5/12 of these genes to be implicated in PCa, while the rest are either involved in other types of tumors or have not yet been shown to be involved in cancer. Briefly, *DICER1* which we found to be upregulated in the tumors compared to normal glands, has previously been reported to be upregulated in PCa (15). Of note, the overexpression of *DICER1* has been suggested to predict poor survival in colorectal cancer (16). As regards *IGF2*, dysregulation has been suggested to be an early change in PCa, and inactivation has been associated with cancer progression (17). *TNRC6A* has been reported to be expressed in 55% of prostatic intraepithelial neoplasia (PIN) and 63.6% of PCa cases (18). Concerning *RBM3*, a high protein expression in PCa has been shown to independently predict a reduced risk of biochemical recurrence and disease progression (19). *PIWIL2* has been reported to be expressed in many tumors including PCa, and has been shown to inhibit apoptosis through the activation of the Stat3/Bcl-xL pathway (20). *TDRD1,* which is known to be specifically expressed in the testis is expressed in both hepatocellular carcinoma and non-tumorous liver tissues (21). *ADARB1*, an editing mediating enzyme, has been detected at reduced RNA levels in brain tumors, and the overexpression of *ADARB1* in a glioblastoma multiforme cell line has been shown to lead to decreased proliferation (22). DGCR8 is a double-cysteine-ligated heme protein (23) and is part of a multi-subunit protein complex known as the microprocessor complex, which has been shown to be necessary and sufficient for processing miRNA precursor RNAs (24). Finally, to our knowledge, there are no cancer studies to date reporting on *HILS1*, *GLMN, TRIM27* and *BC069781*.

In order to gain insight into the potential role in metastasis of the 12 epigenetics-related genes that we identified in primary PCa, we examined the expression profile of the latter genes in the 4 well characterized androgen-sensitive VCaP and LNCaP, and -insensitive PC3 and DU-145 PCa cell lines (Fig. 1). The 4 cell lines were derived from different origins. DU-145, PC3, LNCaP and VCaP cells were derived from brain metastasis, advanced androgen independent bone metastasis, supraclavicular lymph node metastasis and a metastatic lesion to a lumbar vertebral body of a patient with hormone refractory PCa, respectively. Our analysis revealed the expression level for each gene to be different among the 4 cell lines examined (Fig. 1). For example, *TDRD1* was only expressed in the androgen-sensitive PCa cell lines, and not in the insensitive cell lines. *HILS1* and *TNRC6A* were expressed in all the cell lines except in the androgen-sensitive VCaP cell line. *PIWIL2* and *BCO69781* were weakly expressed in all the cell lines examined, whereas *IGF2*, *DICER1*, *ADARB1*, *GLMN*, *TRIM27*, *DGCR8* and *RBM3* were expressed in all 4 cell lines at different levels. The differences in expression pattern of these 12 genes among the 4 cell lines may reflect the different biological properties of each cell line, and the potential role each gene may play at different metastatic sites.

Taken together, in this study, we identify novel epigenetics-related genes in primary PCa tissues, which we believe are highly significant due to the crucial role that epigenetic changes play in PCa. It is likely, based on our gene expression profile data, that some of the genes that we identified may play important roles in primary PCa and metastasis, in primary only, or in metastasis only. Follow-up studies are required to investigate the functional role and the role that the expression of these genes play in the outcome and progression of PCa using tissue microarrays comprised of primary PCa samples, lymph node and distant metastases.

## Figures and Tables

**Table I:** The sequences of primers used in the RT-PCR analysis.

Short Name	Left primer	Right primer
*RBM3*	GCTGCACCGAAGCATCTTAT	CCAAACTTGCCAGAACCAAT
*GLMN*	CAGCAATTGGACACCCTTTC	TGCCCCATATTAAACTGCAA
*BC069781*	TTTGTCAGGCACAGATGCTT	CGATGCTGATTGAGGTTGTG
*TNRC6A*	TTGAATCATGCAGGCCAATA	AGTGCAAAGGGAAAAGCTCA
*TDRD1*	GCTCCACAGCATGTCAAAGA	AGCCCAAATGGCTATTTCCT
*DGCR8*	GGGGGTGAGAGTGCTGATAA	GGGAAATTCAAGGCCTCTTC
*HILS1*	GCTCAAGGTCAAGAGGCAAC	TGCCTCATTAATTGCAGTGG
*TRIM27*	TCAGTGGACGTGACTCTGGA	CCACCTTTTCTGCACACTGA
*DICER1*	AAGGAAGCTGGCAAACAAGA	AAAACGAACCACCAAGTTGC
*PIWIL2*	TCTATGGGGCCATCAAGAAG	CCATCCCGATCACCATTAAC
*IGF2*	TCCTCCCTGGACAATCAGAC	AGAAGCACCAGCATCGACTT
*ADARB1*	ACCTCCACCAAGCTCAGAGA	ATGCTGTGGGGTAAGGTCTG
*GAPDH*	ATGAGGTCCACCACCCTGTT	ATCACTGCCACCCAGAAGAC

**Table II:** Genes upregulated in moderately differentiated tumors compared to normal glands of the peripheral zone.

Short name	Long name	Fold change
*TDRD1*	Tudor domain-containing 1	17.2
*IGF2*	Insulin-like growth factor 2 (somatomedin A); INS-IGF2 readthrough transcript	6.6
*DICER1*	Dicer 1, ribonuclease type III	4.8
*ADARB1*	Adenosine deaminase, RNA-specific	4.6
*HILS1*	Histone linker H1 domain, spermatid-specific 1	3.8
*GLMN*	Glomulin, FKBP associated protein	2.9
*TRIM27*	Tripartite motif-containing 27	2.9

**Table III:** Genes downregulated in moderately differentiated tumors compared to normal glands of the peripheral zone.

Short name	Long name	Fold change
*TNRC6A*	Trinucleotide repeat containing 6A	−2.9
*DGCR8*	DiGeorge syndrome critical region gene 8	−3.4

**Table IV:** Genes upregulated in poorly differentiated tumors compared to normal glands of the peripheral zone.

Short name	Long name	Fold change
*TDRD1*	Tudor domain containing 1	8.5
*ADARB1*	Adenosine deaminase, RNA-specific	5.3
*RBM3*	RNA binding motif (RNP1, RRM) protein 3; hypothetical LOC729275	2.4

**Table V:** Genes downregulated in poorly differentiated tumors compared to normal glands of the peripheral zone.

Short name	Long name	Fold change
*DGCR8*	DiGeorge syndrome critical region gene 8	−3.8
*PIWIL2*	Piwi-like 2 (*Drosophila*)	−3.1
*BC069781*	SMAD family member 1	−3.0
